# Anti-Stem Cell Property of Pterostilbene in Gastrointestinal Cancer Cells

**DOI:** 10.3390/ijms21249347

**Published:** 2020-12-08

**Authors:** Shiori Mori, Shingo Kishi, Kanya Honoki, Rina Fujiwara-Tani, Takuma Moriguchi, Takamitsu Sasaki, Kiyomu Fujii, Shinji Tsukamoto, Hiromasa Fujii, Akira Kido, Yasuhito Tanaka, Yi Luo, Hiroki Kuniyasu

**Affiliations:** 1 Department of Molecular Pathology, Nara Medical University, 840 Shijo-cho, Kashihara 634-8521, Nara, Japan; shi.m.0310@i.softbank.jp (S.M.); nmu6429@yahoo.co.jp (S.K.); rina_fuji@naramed-u.ac.jp (R.F.-T.); takuma1467@i.softbank.jp (T.M.); takamitu@fc4.so-net.ne.jp (T.S.); toto1999-dreamtheater2006-sms@nifty.com (K.F.); 2 Department of Orthopedics, Nara Medical University, 840 Shijo-cho, Kashihara 634-8522, Nara, Japan; kahonoki@naramed-u.ac.jp (K.H.); shinji104@mail.goo.ne.jp (S.T.); hiromasa@naramed-u.ac.jp (H.F.); akirakid@naramed-u.ac.jp (A.K.); yatanaka@naramed-u.ac.jp (Y.T.); 3 Key Laboratory of Neuroregeneration of Jiangsu and Ministry of Education, Co-Innovation Center of Neuroregeneration, Nantong University, Nantong 226001, China

**Keywords:** pterostilbene, cancer, stemness, oxidative stress

## Abstract

Pterostilbene (PTE) is a natural sterbenoid contained in blueberries that has an antioxidant effect. In contrast, PTE also generates oxidative stress in cancer cells and provides an antitumor effect. Here, we examined the potential mechanism of this contrasting effect of PTE using three gastrointestinal cancer cell lines, namely CT26, HT29, and MKN74. PTE showed a dose-dependent inhibition of cell proliferation, sphere-forming ability, and stem cell marker expression in all three cell lines. Furthermore, the cells treated with PTE showed an increase in mitochondrial membrane potential and an increase in mitochondrial oxidative stress and lipid peroxide. Upon concurrent treatment with vitamin E, N-acetyl-L-cysteine, and PTE, the PTE-induced mitochondrial oxidative stress and growth inhibition were suppressed. These findings indicate that PTE induces oxidative stress in cancer cells, suppresses stemness, and inhibits proliferation. These antitumor effects of PTE are considered to be useful in cancer treatment.

## 1. Introduction

Pterostilbene (PTE), a known nutritional ingredient in blueberries, is a natural dimethylated analog of resveratrol (RES) [1]. Compared to RES, which is the same stilbenoid, PTE has a higher bioavailability and a high blood retention for a long duration, which results in stronger effects than RES [2,3]. Due to its antioxidant effects, PTE is expected to be useful for the prevention of carcinogenesis, neurodegenerative diseases, inflammatory diseases, hyperlipidemia, and vascular disorders, as well as for improving diabetes [1,2]. Extensive research has been conducted on the application of PTE to cancer treatment. PTE inhibits cancer growth, anti-apoptotic survival, metastasis, and cancer cell stemness; however, the mechanism has not been completely clarified [4,5].

The mechanism of action of PTE revealed so far is diverse. Its actions in cancer include arrest of cell cycle in S and G2/M phase, induction of apoptosis by reactive oxygen species (ROS) and autophagy, suppression of matrix metalloproteinase-2/-9 expression and sphere formation, and inhibition of cell surface fibronectin polymerization, which diminishes the metastatic potential [6,7,8]. The cancer-related factors and signal transduction pathways that PTE suppresses are also diverse, and include Janus kinase-2/signal transduction and activator of transcription-3, human telomerase reverse transcriptase, c-Myc, mutant epithelial growth factor receptor, protein kinase B (also known as AKT), multiple drug resistance-1, sirtuin-1, DNA methyltransferase, extracellular signal-regulated kinase, cyclin D1, mammalian target of rapamycin, metastasis-associated protein, and nuclear factor-κB [9,10,11,12,13,14,15,16,17]. In addition, PTE activates autophagy, mutated in the Ataxia telangiectasia mutated/Ataxia telangiectasia and Rad3-related/Checkpoint kinase-1/p53 pathway, and sirtuin-1 pathway [13,18,19,20]. Moreover, PTE affects promoter DNA methylation, histone modifications, and microRNAs, resulting in alteration of epigenetic gene expression [20].

The current study aimed to elucidate the central mechanism of such diverse actions of PTE. In particular, PTE has been reported to induce reactive oxidative species (ROS) production, although it is also known to function as an antioxidant; this discrepancy attracted attention [6]. Therefore, we paid special attention to the relationship between the antitumor effect of PTE and ROS in this study.

## 2. Results

### 2.1. Effect of Pterostilbene (PTE) on Cell Proliferation and Stemness in Gastrointestinal Cancer Cells

**Figure 1:**
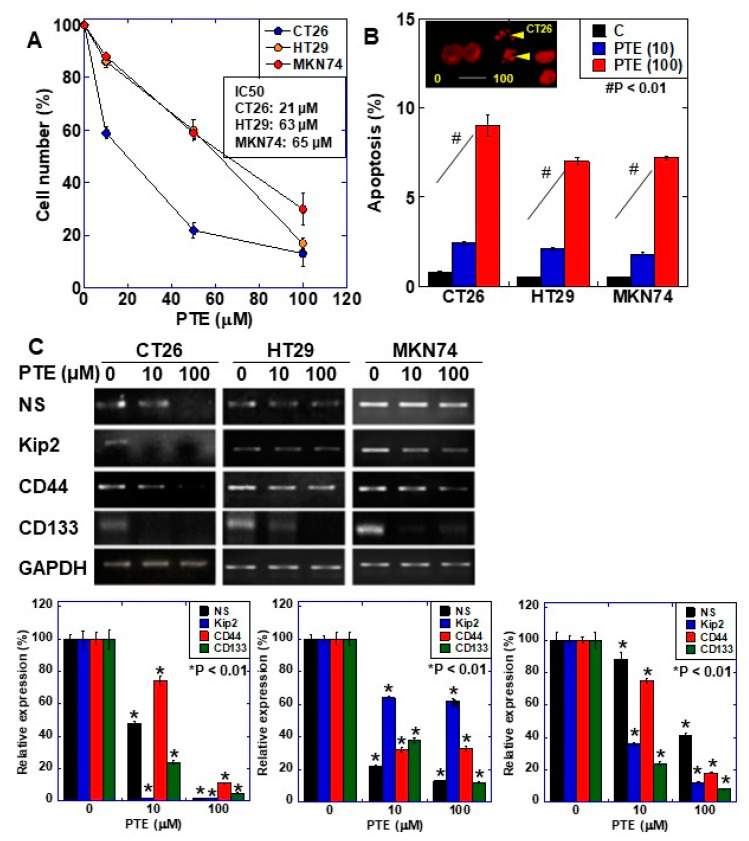
Effect of pterostilbene (PTE) on cell proliferation and expression of stem cell markers in cancer cell lines. (**A**) Cell proliferation was assessed by 3-(4,5-dimethylthiazol-2-yl)-5-(3-carboxymethoxyphenyl)-2-(4-sulfophenyl)-2H-tetrazolium, inner salt (MTS) assay. Cells were treated with PTE for 48 h. (**B**) Apoptosis was assessed by ethidium bromide (EtBr) staining. Insert, CT26 cells treated with PTE for 48 h were stained with EtBr. Arrow head, apoptosis body. Scale bar, 20 µM. (**C**) mRNA expression of stem cell markers; nucleostemin (NS), CD44, Kip2, and CD133 were examined by RT-PCR. Glyceraldehyde 3-phosphate dehydrogenase (GAPDH) was amplified for loading standard. Lower panels were semi-quantification of stem cell marker expression examined by RT-PCR. Error bar, standard deviation from three independent examinations. Statistical difference was calculated by ordinary analysis of variance. * Statistical difference from PTE (0 µM). PTE, pterostilbene; IC50, 50% inhibitory concentration.

**Figure 2:**
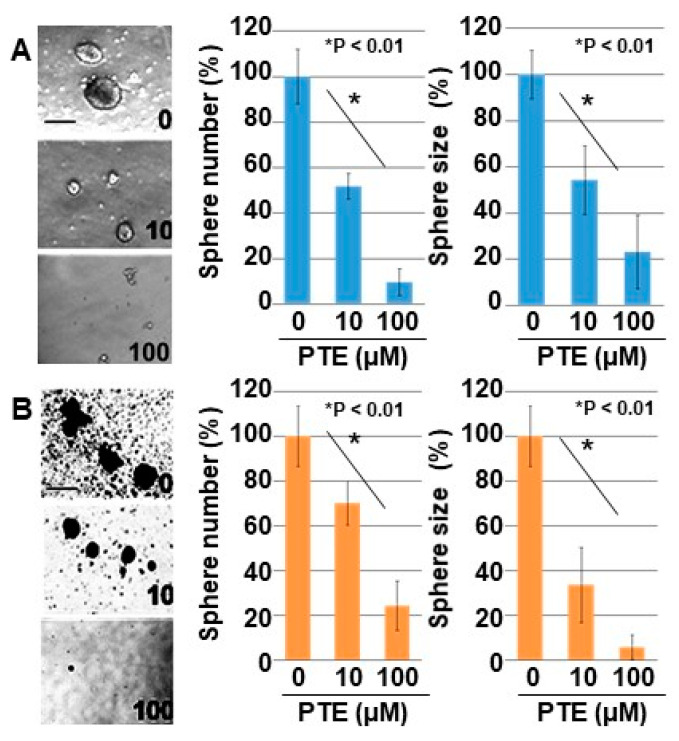
Effect of PTE on sphere formation in cancer cell lines. Sphere formation was examined in 10,000 cells with or without PTE treatment for 7 days. (**A**) CT26 cells. (**B**) HT29 cells. Pictures were images of phase-contrast microscopy. Error bar, standard deviation from three independent examinations. Statistical difference was calculated by ordinary analysis of variance. Scale bar, 50 μm. TE, pterostilbene.

The effect of PTE on cell proliferation was examined using three gastrointestinal cancer cell lines, namely HT29, MKN74, and CT26 (Figure 1A). Dose-dependent growth inhibition was observed in all three cell lines, including at the low concentration of 10 μM of PTE. Next, we examined the gene expression of four types of stem cell markers to evaluate the effect of PTE on stemness (Figure 1B). Expression of the four markers decreased in a dose-dependent manner in all cell lines. The effect of PTE on stemness was also examined by sphere assay (Figure 2). In both CT26 cells (Figure 2A) and HT29 cells (Figure 2B), sphere formation was suppressed in terms of the number and size in a dose-dependent manner.

### 2.2. Effect of PTE on Generation of Reactive Oxygen Species (ROS)

**Figure 3:**
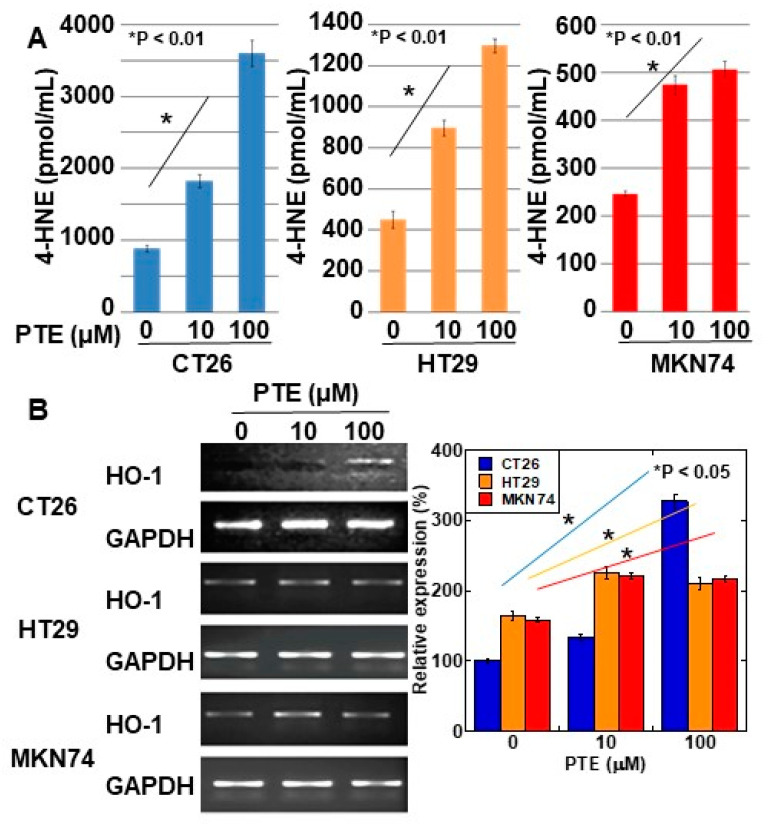
Effect of PTE on oxidative stress in cancer cell lines. (**A**) 4-hydroxynonenal (4-HNE) levels were measured by enzyme-linked immunosorbent assay (ELISA). Cells were treated with PTE for 48 h. (**B**) mRNA expression of heme oxygenase-1 (HO-1) was examined by RT-PCR. GAPDH was amplified as a loading standard. Right panel was semi-quantification of HO-1 expression examined by RT-PCR. Error bar, standard deviation from three independent examinations. Statistical difference was calculated by ordinary analysis of variance. PTE, pterostilbene; HNE. Hydroxynonenal; HO, heme oxygenase.

Intracellular levels of 4-hydroxynonenal (4-HNE) were determined for evaluating ROS generation by PTE treatment (Figure 3A). In all three cell lines, 4-HNE concentration increased in a PTE dose-dependent manner. Furthermore, we investigated the gene expression of heme oxygenase (HO)-1, which is known to be a rescue protein against oxidative stress [21,22] (Figure 3B). In CT26 cells, a high concentration of PTE (100 μM) induced heme oxygenase-1 (HO-1) expression; however, in HT29 and MKN74 cells, low- and high-concentrations of PTE (10 and 100 μM, respectively) induced HO-1 expression.

**Figure 4:**
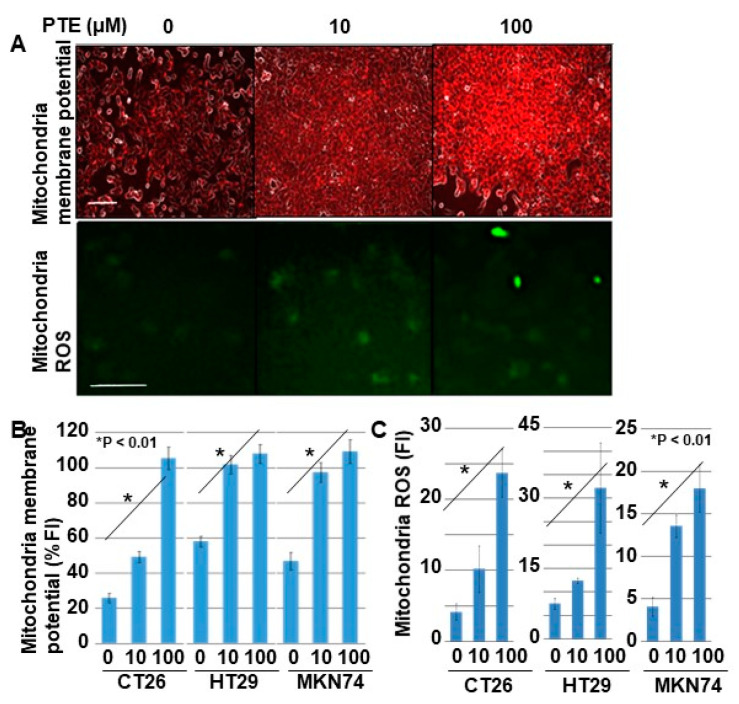
Effect of PTE on mitochondrial function in cancer cells. Mitochondrial membrane voltage and mitochondrial reactive oxidative species (ROS) were examined by tetramethyl rhodamine (TMRE) and dihydrorhodamine (DHR), respectively. Cells were treated with PTE for 48 h. (**A**) Fluorescence images of TMRE and DHR. TMRE image was merged with phase-contrasted image. Scale bar, 25 μm. (**B**,**C**) Semi-quantification of mitochondrial membrane potential (TMRE) and mitochondrial ROS (DHR), respectively. Error bar, standard deviation from three independent examinations. Statistical difference was calculated by ordinary analysis of variance. PTE, pterostilbene; TMRE, tetramethyl rhodamine; ROS, reactive oxidative species; DHR, dihydrorhodamine 123; FI, fluorescence intensity.

Next, we examined the effect of PTE on mitochondrial membrane potential and mitochondrial ROS generation (Figure 4). The mitochondrial membrane potential of all cell lines increased in a PTE-dose-dependent manner (Figure 4A,B). Furthermore, ROS derived from mitochondria also increased in a PTE-dose-dependent manner (Figure 4A,C).

**Figure 5:**
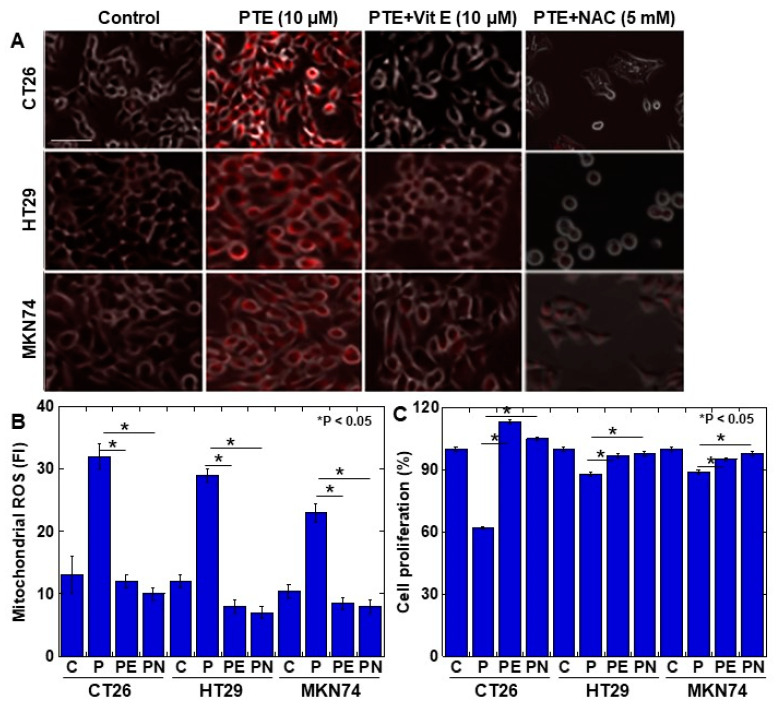
Effect of vitamin E on PTE-induced oxidative stress in cancer cell lines. Cells were treated with or without PTE (10 μM) and/or vitamin E (10 μM) and/or N-acetyl-L-cysteine (NAC) (5 mM) for 48 h. (**A**) Mitochondrial ROS was assessed by MitoROS (red color). MitoROS image was merged with phase-contrasted image. (**B**) Cell proliferation was assessed by MTS. (**C**) Semi-quantification of mitochondrial ROS. C, control; P, PTE; PE, PTE+vitamin E; PN, PTE+NAC. Error bar, standard deviation from three independent examinations. Statistical difference was calculated by ordinary analysis of variance. Scale bar, 25 μm. PTE, pterostilbene; ROS, reactive oxidative species; FI, fluorescence intensity; NAC, N-acetyl-L-cysteine; MTS, 3-(4,5-dimethylthiazol-2-yl)-5-(3-carboxymethoxyphenyl)-2-(4-sulfophenyl)-2H-tetrazolium.

Finally, to examine whether PTE-induced ROS generation causes cytotoxicity, cells were treated with vitamin E or N-acetyl-L-cysteine (NAC) to suppress ROS generation (Figure 5). The generation of mitochondrial ROS was almost completely suppressed to the control levels by vitamin E and NAC (Figure 5A,B). The concurrent treatment with PTE and vitamin E or NAC showed that the PTE-mediated inhibition of cell proliferation was rescued by vitamin E and NAC (Figure 5C).

## 3. Discussion

In our study, PTE treatment resulted in a decrease in stemness along with an increase in mitochondrial ROS production in all three gastrointestinal cancer cell lines that were tested. Previously, it was reported that PTE suppresses the expression of CD44, c-Myc, and cyclin D, and hedgehog/AKT signal, which preferentially damages cancer stem cells [23]. However, the mechanism is not yet fully understood. RES, which has a similar structure to PTE, suppresses Wnt/beta-catenin and reduces stemness in breast cancer cells [5]. In addition, RES has been reported to promote argonaute2 expression and suppress tumor stem cells by inducing tumor suppressor microRNA expression [24]. PTE has been suggested to have a similar mechanism. In addition, PTE suppresses the stat3 pathway in HeLa cells and reduces stemness, and it displays a stronger effect than RES [6].

In our study, PTE induced strong ROS production in all three cancer cells. While low concentrations of ROS promote stemness and epithelial-mesenchymal transition in cancer cells [25,26], high concentrations induce apoptosis [27,28]. PTE has been suggested to induce high levels of ROS, leading to cell death, especially in cancer stem cells that show undifferentiated properties, but not in differentiated cells. This study has been conducted in order to prove the suppression of tumor cells by low concentration of PTE, such as supplements. The antitumor effect of even 10 μM of PTE suggests that the safe and effective therapeutic application of PTE is possible.

We found that PTE markedly enhanced mitochondrial ROS production. To date, it has been reported that PTE causes a decrease in mitochondrial membrane potential, an increase in oxidative stress, and an alteration in mitochondrial calcium ion concentration in cancer cells, resulting in induction of apoptosis [14,29,30,31]. The mechanism of PTE on the mitochondrial membrane potential is not clear. RES, the same stilbenoid as PTE, causes voltage-dependent anion channel (VDAC) dephosphorylation in cardiomyocytes [32], inhibits mitochondrial membrane permeability, and suppresses mitochondrial injury and apoptosis [32]. On the contrary, VDAC phosphorylation enhances mitochondrial permeability and induces apoptosis [33].

It is possible that PTE may elicit a similar effect to that of RES on VDAC-mediated mitochondrial membrane potential. In our study, mitochondrial potential was enhanced by PTE, which contradicts many reports [14,34]. Unlike apoptosis, in which the mitochondrial membrane potential decreases, the involvement of ferroptosis can explain the increase in mitochondrial membrane potential. In ferroptosis, the inducer elastin opens VDAC, promotes mitochondrial hyperpolarization, and increases ROS production [35,36]. Induction of cell death in cancer cells by PTE is reported to be oxidative stress-induced apoptosis [14,29,31]. However, PTE-induced cell death is only partially rescued by apoptosis-inhibiting peptides, and mechanisms other than apoptosis are thought to be involved [37]. From these, it is suggested that cell death by PTE might be a mixture of apoptosis and ferroptosis.

Notably, ferroptosis is induced by lipid peroxide [38], and our data also show an increase in lipid peroxide 4-HNE upon PTE treatment. Furthermore, ferroptosis is suppressed by the conversion of peroxyl radicals to peroxides by vitamin E and coenzyme Q10 [38]. In our experiment, vitamin E and NAC reduced the cytotoxicity of PTE. Vitamin E removes singlet oxygen and hydroxyl radicals, and suppresses lipid peroxide production, whereas NAC is a precursor of glutathione and broadly regulates redox. Both antioxidants suppressed PTE-induced cytotoxicity. We determined ROS with 4-HNE, dihydrorhodamine (DHR) 123, and mitoROS. 4-HNE, DHR123, and mitoROS detect lipid peroxide, hydrogen peroxide, and superoxide, respectively, and both were increased by PTE and decreased by vitamin E and NAC. These results suggest that PTE produces various types of ROS.

The strongest inducer of ferroptosis is elastin, but cysteine deficiency also induces ferroptosis via lipid peroxidation [35,39]. PTE promotes the dissociation of Kelch-like ECH-associated protein (Keap)-1 and NF-E2-related factor 2 (Nrf2), and promotes the expression of HO-1 and NAD(P)H:quinine oxidoreductase, glutamate-cysteine ligase catalytic subunit, and glutamate-cysteine ligase modifier, which are downstream target genes [40]. Our data also showed enhanced HO-1 expression upon PTE treatment, suggesting mediation of the Keap-1/Nrf2 pathway. Glutathione-SH consumption due to glutamate-cysteine ligase activation and increased ROS production might cause a decrease in intracellular cysteine and induce ferroptosis.

In our study, PTE showed a strong inhibitory effect on cancer stem cells. PTE suppressed expression stemness marker genes and sphere formation. Thus, PTE is expected to be effective for targeting cancer stem cells [5]. However, it is important to consider the potential effects of PTE on normal cells. PTE activates the Sirtuin-1/Forkhead boxO1/p53 pathway in skeletal muscle cells and reduces oxidative stress damage and mitochondrial dysfunction associated with ischemia reperfusion [19]. PTE induces manganese-dependent superoxide dismutase expression through extracellular-signal-regulated kinase-5/Histone deacetylase-5 pathway and enhances oxidative stress tolerance in vascular endothelial cells [41]. Oxidative stress is also suppressed in hepatocytes [42]. HO-1 expression induced by PTE reduces the oxidative stress damage associated with ischemia reperfusion in nerve cells [43]. Furthermore, PTE suppresses adipocyte differentiation of 3T3L cells and retains its stemness [44]. As described above, PTE acts reducing oxidative stress and protects stem cells in normal tissues, exhibiting a function opposite to that observed in cancer tissues.

As a factor that causes a difference in the action of PTE, as described above, on cancer and normal tissue, qualitative differences in mitochondria, which are the major organelles of ROS production, are emphasized. It is considered that the mitochondria in cancer cells are aberrant. An imbalance in mitochondrial electron transport protein expression is seen in many cases of liver cancer [45]. We also previously reported that the promotion of oxidative phosphorylation by lauric acid induces high levels of oxidative stress [46]. Lauric acid, a medium-chain fatty acid, enhances oxidative phosphorylation, which produces ROS in cancer cells with imbalance in mitochondrial electron transport protein expression [46]. In contrast, normal cells, such as lymphocytes and myocardial cells, which carry mitochondrial electron transport proteins, are not induced in ROS differently from cancer cells [46,47]. These findings suggest that PTE might provide ROS in cancer cells with a mutated electron transport system, whereas PTE might reduce ROS in normal cells with a normal electron transport system. Mutations in mitochondrial DNA in cancer cells are associated with suppression of oxidative phosphorylation and increased oxidative stress [48,49]. In particular, among mitochondrial DNA, mutations in MtCOI and MtND6 affect the assembly of respiratory complexes [49].

HO-1 expression level is associated with resistance to ROS [22]. In our data, the constitutive expression level of HO-1 is higher in HT29 and MKN74 cells than that in CT26 cells and HO-1 expression was induced by low and high concentrations (10 and 100 μM, respectively) of PTE in the two cell lines. In contrast, CT26 cells showed low constitutive expression of HO-1 and were induced at high concentration of PTE. Interestingly, HO-1 expression level was associated with mitochondrial membrane potential. Nrf2 was linked to mitochondrial membrane potential [50] and Nrf2 induces HO-1 expression [51]. As discussed earlier, PTE is thought to increase mitochondrial membrane potential in a ferroptosis-related situation, suggesting that it might induce HO-1 expression via Nerf2 activation.

Here, our study confirmed that PTE exerts an excellent antitumor effect, suggesting the involvement of ferroptosis in its mechanism. Especially, the antitumor effect shown in such low-dose PTE (10 μM) suggests that safe and effective therapeutic application of PTE is possible. In the future, it is expected that a more detailed elucidation of its mechanism of action will enable the effective application of PTE to cancer treatment.

## 4. Materials and Methods

### 4.1. Cell Lines and Reagents

HT29 human carcinoma cell lines were purchased from Dainihon Pharmacy Co. (Tokyo, Japan). MKN74 human gastric carcinoma cell line was obtained from the Japanese Collection of Research Bioresources (JCRB; Osaka, Japan). CT26 murine colon carcinoma cell line was gifted by Professor I. J. Fidler (MD Anderson Cancer Center, TX, USA). Cells were cultured in Dulbecco’s modified Eagle’s medium supplemented with 10% fetal bovine serum at 37 °C in 5% CO2. PTE (Tokyo Chemical Industry Co., Ltd., Tokyo, Japan) and vitamin E (Sigma-Aldrich Inc., St. Louis, MO, USA), N-acetyl-L-cysteine (NAC, Sigma) were purchased. Cells were treated with PTE (10, 50, and 100 μM).

Apoptosis was assessed by staining with EtBr (Sigma). From 200 cells observed under fluorescence microscopy, apoptotic body was counted.

Tetramethyl rhodamine (TMRE), dihydrorhodamine 123 (DHR), and mitoROS were purchased (Sigma). Semi-quantification of fluorescence images was performed by using the luminance measurement mode of fluorescence microscope (Keyence Corp., Osaka, Japan).

### 4.2. MTS (3-(4,5-Dimethylthiazol-2-yl)-5-(3-Carboxymethoxyphenyl)-2-(4-Sulfophenyl)-2H-Tetrazolium) Assay

MTS assays were performed using a Celltiter 96 Aqueous One Solution Cell Proliferation Assay kit (Promega Biosciences, Inc., San Louis Obispo, CA, USA). The plates were read on a multiscan FC microplate photometer at 490 nm. The MTS value in cells cultured with the control oligonucleotide was used as the control.

### 4.3. Sphere Assay

Cells (10,000 cells per well) were seeded on uncoated bacteriological 35-mm dish (Coning Inc., Coning, NY, USA) with 3D Tumorsphere Medium XF (Sigma). Cells were cultured with or without PTE (10, 50, 100 μM). After 7 days, sphere images were captured on a computer and the sphere size was measured using NIH ImageJ software (version 1.52, NIH, Bethesda, MD, USA). We counted the number of all spheres in the culture well. The sphere size designated was a mean of all spheres in the well.

### 4.4. Reverse Transcription-Polymerase Chain Reaction (RT-PCR)

To assess human and murine mRNA expression, RT-PCR was performed with 0.5 µg total RNA extracted using a RNeasy kit from the three cell lines (Qiagen, Germantown, MD, USA). The primer sets are listed in Table 1 and were synthesized by Sigma Genosys (Ishikari, Japan). PCR products were electrophoresed using a 2% agarose gel and stained with ethidium bromide. The GAPDH mRNA was also amplified for use as an internal control. Semi-quantification of RT-PCR was performed by using ImageJ (NIH).

### 4.5. Protein Extraction

Whole-cell lysates were prepared as previously described using 0.1% SDS-added RIPA-buffer (Thermo Fisher Scientific, Tokyo, Japan) [52]. Protein assay was performed using a Protein Assay Rapid Kit (Wako Pure Chemical Corporation, Osaka, Japan).

### 4.6. Enzyme-Linked Immunosorbent Assay (ELISA) and Fluorometric Assay

ELISA kit was used to measure the concentration of 4-hydroxynonenal (HNE) (Abcam, Cambridge, MA, USA). The assay was performed according to the manufacturer’s instructions, and whole-cell lysates were used for the measurements.

### 4.7. Statistical Analysis

Statistical significance was calculated using a two-tailed Fisher’s exact test, an ordinary analysis of variance (ANOVA), and InStat software (version 3.0, GraphPad, Los Angeles, CA, USA). A two-sided *p*-value of < 0.05 was considered to indicate statistical significance.

## Figures and Tables

**Table 1:** Primer sets for RT-PCR.

Gene Name	Species	Gene Bank ID	Primer Sequence
*cd44*	mouse	M27130.1	TGGATCCGAATTAGCTGGAC
			AGCTTTTTCTTCTGCCCACA
*cd133*	mouse	BC028286.1	GAAAAGTTGCTCTGCGAACC
			TCTCAAGCTGAAAAGCAGCA
*kip2*	mouse	U22399.1	CTGACCTCAGACCCAATTCC
			CTCAGAGACCGGCTCAGTTC
*ho-1*	mouse	NM_010442.2	CACGCATATACCCGCTACCT
			CCAGAGTGTTCATTCGAGCA
*Nucleostemin*	mouse	BC037996.1	ATGTGGGGAAAAGCAGTGTC
			TGGGGGAGTTACAAGGTGAG
*gapdh*	mouse	NM_001289726.1	AACTTTGGCATTGTGGAAGG
			ACACATTGGGGGTAGGAACA
*CD44*	human	FJ216964.1	AAGGTGGAGCAAACACAACC
			AGCTTTTTCTTCTGCCCACA
*CD133*	human	BC012089.1	TTGTGGCAAATCACCAGGTA
			TCAGATCTGTGAACGCCTTG
*Kip2*	human	AB012955.1	CCCGGCTCACTAAGTCAGAG
			AGATCCGGATGTGGAAAGTG
*Nucleostemin*	human	BC001024.2	ATTGCCAACAGTGGTGTTCA
			AATGGCTTTGCTGCAAGTTT
*GAPDH*	human	BC025925.1	GAGTCAACGGATTTGGTCGT
			TTGATTTTGGAGGGATCTCG

## Abbreviations

PTE	Pterostilbene
RES	Resveratrol
ROS	Reactive oxygen species
4-HNE	4-hydroxynonenal
HO-1	Heme oxygenase-1
VDAC	Voltage-dependent anion channel
Keap-1	Kelch-like ECH-associated protein 1
Nrf-2, NF-E2	NF-E2-related factor 2
NS	Nucleaostemine

## References

[1] McCormack D., McFadden D. (2013). A Review of Pterostilbene Antioxidant Activity and Disease Modification. Oxidative Med. Cell. Longev..

[2] Kosuru R., Rai U., Prakash S., Singh A., Singh S. (2016). Promising therapeutic potential of pterostilbene and its mechanistic insight based on preclinical evidence. Eur. J. Pharmacol..

[3] Ma Z., Zhang X., Xu L., Liu D., Di S., Li W., Zhang J., Zhang H., Li X., Han J. (2019). Pterostilbene: Mechanisms of its action as onc ostatic agent in cell models and in vivo studies. Pharmacol. Res..

[4] McCormack D., McFadden D. (2012). Pterostilbene and Cancer: Current Review. J. Surg. Res..

[5] Dandawate P.R., Subramaniam D., A Jensen R., Anant S. (2016). Targeting cancer stem cells and signaling pathways by phytochemicals: Novel approach for breast cancer therapy. Semin. Cancer Biol..

[6] Shin H.J., Han J.M., Choi Y.S., Jung H.J. (2020). Pterostilbene Suppresses both Cancer Cells and Cancer Stem-Like Cells in Cervical Cancer with Superior Bioavailability to Resveratrol. Molecules.

[7] Tsai H.-Y., Ho C.-T., Chen Y.-K. (2017). Biological actions and molecular effects of resveratrol, pterostilbene, and 3′-hydroxypterostilbene. J. Food Drug Anal..

[8] Wang Y.-J., Lin J.-F., Cheng L.-H., Chang W.-T., Kao Y.-H., Chang M.-M., Wang B.-J., Cheng H.-C. (2017). Pterostilbene prevents AKT-ERK axis-mediated polymerization of surface fibronectin on suspended lung cancer cells independently of apoptosis and suppresses metastasis. J. Hematol. Oncol..

[9] Wen W., Lowe G., Roberts C.M., Finlay J., Han E.S., Glackin C.A., Dellinger T.H. (2018). Pterostilbene Suppresses Ovarian Cancer Growth via Induction of Apoptosis and Blockade of Cell Cycle Progression Involving Inhibition of the STAT3 Pathway. Int. J. Mol. Sci..

[10] Liu Y., Wang L., Wu Y., Lv C., Li X., Cao X., Yang M., Feng D., Luo Z. (2013). Pterostilbene exerts antitumor activity against human osteosarcoma cells by inhibiting the JAK2/STAT3 signaling pathway. Toxicology.

[11] Daniel M., Tollefsbol T.O. (2018). Pterostilbene down-regulates hTERT at physiological concentrations in breast cancer cells: Potentially through the inhibition of cMyc. J. Cell. Biochem..

[12] Bracht J.W.P., Karachaliou N., Berenguer J., Pedraz-Valdunciel C., Filipska M., Codony-Servat C., Codony-Servat J., Rosell R. (2019). Osimertinib and pterostilbene in EGFR-mutation-positive non-small cell lung cancer (NSCLC). Int. J. Biol. Sci..

[13] Chang H.P., Lu C.C., Chiang J.H., Tsai F.J., Juan Y.N., Tsao J.W., Chiu H.Y., Yang J.S. (2018). Pterostilbene modulates the suppression of multidrug resistance protein 1 and triggers autophagic and apoptotic mechanisms in cisplatin-resistant human oral cancer CAR cells via AKT signaling. Int. J. Oncol..

[14] Bin W.H., Da L.H., Xue Y., Jing B. (2018). Pterostilbene (3’,5’-dimethoxy-resveratrol) exerts potent antitumor effects in HeLa human cervical cancer cells via disruption of mitochondrial membrane potential, apoptosis induction and targeting m-TOR/PI3K/Akt signalling pathway. J. Buon.

[15] Kala R., Shah H.N., Martin S.L., Tollefsbol T.O. (2015). Epigenetic-based combinatorial resveratrol and pterostilbene alters DNA damage response by affecting SIRT1 and DNMT enzyme expression, including SIRT1-dependent γ-H2AX and telomerase regulation in triple-negative breast cancer. BMC Cancer.

[16] Wakimoto R., Ono M., Takeshima M., Higuchi T., Nakano S. (2017). Differential Anticancer Activity of Pterostilbene Against Three Subtypes of Human Breast Cancer Cells. Anticancer. Res..

[17] Dhar S., Kumar A., Zhang L., Rimando A.M., Lage J.M., Lewin J.R., Atfi A., Zhang X., Levenson A.S. (2016). Dietary pterostilbene is a novel MTA1-targeted chemopreventive and therapeutic agent in prostate cancer. Oncotarget.

[18] Lee H., Kim Y., Jeong J.H., Ryu J.-H., Kim W. (2016). ATM/CHK/p53 Pathway Dependent Chemopreventive and Therapeutic Activity on Lung Cancer by Pterostilbene. PLoS ONE.

[19] Cheng Y., Di S., Fan C., Cai L., Gao C., Jiang P., Hu W., Ma Z., Jiang S., Dong Y. (2016). SIRT1 activation by pterostilbene attenuates the skeletal muscle oxidative stress injury and mitochondrial dysfunction induced by ischemia reperfusion injury. Apoptosis.

[20] Seo E.J., Fischer N., Efferth T. (2018). Phytochemicals as inhibitors of NF-κB for treatment of Alzheimer’s disease. Pharmacol. Res..

[21] Waza A.A., Hamid Z., Ali S., Bhat S.A., Bhat M.A. (2018). A review on heme oxygenase-1 induction: Is it a necessary evil. Inflamm. Res..

[22] Sasaki T., Yoshida K., Kondo H., Ohmori H., Kuniyasu H. (2005). Heme oxygenase-1 accelerates protumoral effects of nitric oxide in cancer cells. Virchows. Archiv..

[23] Wu C.-H., Hong B.-H., Ho C.-T., Yen G.-C. (2015). Targeting Cancer Stem Cells in Breast Cancer: Potential Anticancer Properties of 6-Shogaol and Pterostilbene. J. Agric. Food Chem..

[24] Hagiwara K., Kosaka N., Yoshioka Y., Takahashi R.-U., Takeshita F., Ochiya T. (2012). Stilbene derivatives promote Ago2-dependent tumour-suppressive microRNA activity. Sci. Rep..

[25] Wang Y.L.A.F.H.S.Z., Li Y., Sarkar F.H. (2010). Signaling Mechanism(S) of Reactive Oxygen Species in Epithelial-Mesenchymal Transition Reminiscent of Cancer Stem Cells in Tumor Progression. Curr. Stem Cell Res. Ther..

[26] Emanuele S., D’Anneo A., Calvaruso G., Cernigliaro C., Giuliano M., Lauricella M. (2018). The Double-Edged Sword Profile of Redox Signaling: Oxidative Events as Molecular Switches in the Balance between Cell Physiology and Cancer. Chem. Res. Toxicol..

[27] Grasso D., Zampieri L.X., Capelôa T., Van de Velde J.A., Sonveaux P. (2020). Mitochondria in cancer. Cell Stress.

[28] Chaudhari P., Ye Z., Jang Y.-Y. (2014). Roles of Reactive Oxygen Species in the Fate of Stem Cells. Antioxidants Redox Signal..

[29] Dong J., Guo H., Chen Y. (2016). Pterostilbene induces apoptosis through caspase activation in ovarian cancer cells. Eur. J. Gynaecol. Oncol..

[30] Kong Y., Chen G., Xu Z., Yang G., Li B., Wu X., Xiao W., Xie B., Hu L., Sun X. (2016). Pterostilbene induces apoptosis and cell cycle arrest in diffuse large B-cell lymphoma cells. Sci. Rep..

[31] Alosi J.A., McDonald D., Schneider J.S., Privette A.R., McFadden D. (2010). Pterostilbene Inhibits Breast Cancer in vitro through Mitochondrial Depolarization and Induction of Caspase-Dependent Apoptosis. J. Surg. Res..

[32] Tian M., Xie Y., Meng Y., Ma W., Tong Z., Yang X., Lai S., Zhou Y., He M., Liao Z. (2019). Resveratrol protects cardiomyocytes against anoxia/reoxygenation via dephosphorylation of VDAC1 by Akt-GSK3 β pathway. Eur. J. Pharmacol..

[33] Chen Y., Craigen W.J., Riley D.J. (2009). Nek1 regulates cell death and mitochondrial membrane permeability through phosphorylation of VDAC1. Cell Cycle.

[34] Storniolo C.E., Moreno J.J. (2019). Resveratrol Analogs with Antioxidant Activity Inhibit Intestinal Epithelial Cancer Caco-2 Cell Growth by Modulating Arachidonic Acid Cascade. J. Agric. Food Chem..

[35] Gao M., Yi J., Zhu J., Minikes A.M., Monian P., Thompson C.B., Jiang X. (2019). Role of Mitochondria in Ferroptosis. Mol. Cell.

[36] Dehart D.N., Fang D., Heslop K., Li L., Lemasters J.J., Maldonado E.N. (2018). Opening of voltage dependent anion channels promotes reactive oxygen species generation, mitochondrial dysfunction and cell death in cancer cells. Biochem. Pharmacol..

[37] Hsiao P.-C., Chou Y.-E., Tan P., Lee W.-J., Yang S.-F., Chow J.-M., Chen H.-Y., Lin C.-H., Lee L.-M., Chien M.-H. (2014). Pterostilbene Simultaneously Induced G0/G1-Phase Arrest and MAPK-Mediated Mitochondrial-Derived Apoptosis in Human Acute Myeloid Leukemia Cell Lines. PLoS ONE.

[38] Conrad M., Kagan V.E., Bayir H., Pagnussat G.C., Head B., Traber M.G., Stockwell B.R. (2018). Regulation of lipid peroxidation and ferroptosis in diverse species. Genes Dev..

[39] Ždralević M., Vučetić M., Daher B., Marchiq I., Parks S.K., Pouysségur J. (2018). Disrupting the ‘Warburg effect’ re-routes cancer cells to OXPHOS offering a vulnerability point via ‘ferroptosis’-induced cell death. Adv. Biol. Regul..

[40] Yang H., Hua C., Yang X., Fan X., Song H., Peng L., Ci X. (2020). Pterostilbene prevents LPS-induced early pulmonary fibrosis by suppressing oxidative stress, inflammation and apoptosis in vivo. Food Funct..

[41] Gan W., Dang Y., Han X., Ling S., Duan J., Liu J., Xu J.W. (2016). ERK5/HDAC5-mediated, resveratrol-, and pterostilbene-induced expression of MnSOD in human endothelial cells. Mol. Nutr. Food Res..

[42] Dong G.-Z., Lee Y.-I., Jeong J.H., Zhao H.-Y., Jeon R., Lee H.J., Ryu J.-H. (2015). Stilbenoids from Rheum undulatum Protect Hepatocytes against Oxidative Stress through AMPK Activation. Phytotherapy Res..

[43] Yang Y., Wang J., Li Y., Fan C., Jiang S., Zhao L., Di S., Xin Z., Wang B., Wu G. (2016). HO-1 Signaling Activation by Pterostilbene Treatment Attenuates Mitochondrial Oxidative Damage Induced by Cerebral Ischemia Reperfusion Injury. Mol. Neurobiol..

[44] Seo Y.-J., Kim K.-J., Koh E.-J., Choi J., Lee B.-Y. (2017). Anti-adipogenesis mechanism of pterostilbene through the activation of heme oxygenase-1 in 3T3-L1 cells. Phytomedicine.

[45] Lee Y.-K., Jee B.A., Kwon S.M., Yoon Y.-S., Xu W.G., Wang H.-J., Wang X.W., Thorgeirsson S.S., Lee J.-S., Woo H.G. (2015). Identification of a mitochondrial defect gene signature reveals NUPR1 as a key regulator of liver cancer progression. Hepatology.

[46] Kadochi Y., Mori S., Fujiwara-Tani R., Luo Y., Nishiguchi Y., Kishi S., Fujii K., Ohmori H., Kuniyasu H. (2017). Remodeling of energy metabolism by a ketone body and medium-chain fatty acid suppressed the proliferation of CT26 mouse colon cancer cells. Oncol. Lett..

[47] Nukaga S., Mori T., Miyagawa Y., Fujiwara-Tani R., Sasaki T., Fujii K., Mori S., Goto K., Kishi S., Nakashima C. (2020). Combined administration of lauric acid and glucose improved cancer-derived cardiac atrophy in a mouse cachexia model. Cancer Sci..

[48] Cruz-Bermúdez A., Vicente-Blanco R.J., Gonzalez-Vioque E., Provencio M., Fernández-Moreno M.Á., Garesse R. (2016). Spotlight on the relevance of mtDNA in cancer. Clin. Transl. Oncol..

[49] Księżakowska-Łakoma K., Żyła M., Wilczyński J.R. (2014). Mitochondrial dysfunction in cancer. Prz. Menopauzalny.

[50] Jung K.A., Lee S., Kwak M.K. (2017). NFE2L2/NRF2 Activity Is Linked to Mitochondria and AMP-Activated Protein Kinase Signaling in Cancers Through miR-181c/Mitochondria-Encoded Cytochrome c Oxidase Regulation. Antioxid. Redox Signal..

[51] Loboda A., Damulewicz M., Pyza E., Jozkowicz A., Dulak J. (2016). Role of Nrf2/HO-1 system in development, oxidative stress response and diseases: An evolutionarily conserved mechanism. Cell. Mol. Life Sci..

[52] Kuniyasu H., Oue N., Wakikawa A., Shigeishi H., Matsutani N., Kuraoka K., Ito R., Yokozaki H., Yasui W. (2002). Expression of receptors for advanced glycation end-products (RAGE) is closely associated with the invasive and metastatic activity of gastric cancer. J. Pathol..

